# Chronic ileocolic intussusception due to transmural infiltration of diffuse large B cell lymphoma in a 14-year-old boy: a case report

**DOI:** 10.1186/s40064-015-1157-6

**Published:** 2015-07-22

**Authors:** Ryuta Saka, Takashi Sasaki, Ikuo Matsuda, Satoko Nose, Masafumi Onishi, Tetsurou Fujino, Hideki Shimomura, Yoshitoshi Otsuka, Noriko Kajimoto, Seiichi Hirota, Takaharu Oue

**Affiliations:** Department of Pediatric Surgery, Hyogo College of Medicine, 1-1 Mukogawa-cho, Nishinomiya, Hyogo 6638501 Japan; Department of Surgical Pathology, Hyogo College of Medicine, Nishinomiya, Japan; Department of Pediatrics, Hyogo College of Medicine, Nishinomiya, Japan

**Keywords:** Chronic intussusception, Lymphoma

## Abstract

Chronic intussusception, defined as intussusception continuing over 14 days, is rare in children. We herein report a case of chronic ileocolic intussusception caused by the transmural infiltration of diffuse large B cell lymphoma in a 14-year-old boy. The patient had been suffering from anorexia and intermittent abdominal pain for 5 weeks, during which his body weight decreased by around 7 kg. Upon admission to our hospital, ultrasonography and enhanced computed tomography (CT) of the abdomen showed ileocolic intussusception. A retrospective examination of abdominal CT led us to suspect that the intussusception had initially appeared 5 weeks before admission, presumably coinciding with the beginning of the patient’s abdominal symptoms. Since hydrostatic reduction was unsuccessful, laparotomy was performed, which showed unreducible ileocolic intussusception with a marked edematous ileum and mesentery. Ileocecal resection without lymph node dissection was carried out, and a histological examination of the resected specimen revealed the transmural infiltration of diffuse large B-cell lymphoma of the terminal ileum. The patient’s postoperative course was uneventful, and adjuvant chemotherapy was administered. This case illustrates the diagnostic challenges of confirming ‘chronic’ intussusception in older children.

## Background

Intussusception is usually an acute condition and is readily diagnosed based on a typical pattern of abdominal pain, “currant jelly” bloody stools and vomiting in children under 2 years old (Schulman et al. 1998). ‘Chronic’ intussusception is a rare entity defined as intussusception continuing over 14 days (Rees and Lari 1976). Chronic intussusception is non-strangulated and incompletely obstructing. Therefore, both the symptoms and causes of ‘chronic’ intussusception may differ from those of ‘acute’ cases. We herein report a case of chronic ileocolic intussusception resulting from the transmural infiltration of diffuse large B-cell lymphoma.

## Case report

A 14-year-old boy showing paroxysmal kinesigenic dyskinesia was referred to our hospital. He had been suffering from anorexia, nausea, abdominal pain and weight loss of 7 kg for 5 weeks. No bilious emesis was reported. At a previous clinic, he was treated as having enterocolitis or anorexia nervosa, without an improvement. On admission, he looked pale and exhibited fatigue. A physical examination showed that the abdomen was not distended, although a mass, with tenderness, was palpable in the lower abdomen. His abdominal pain was colicky, intermittent and severe at times. Although diarrhea and “currant jelly” bloody stools were not present at that time, the stool was positive for occult blood. A culture of the stools was not remarkable, and the results of laboratory tests of the blood (including lactate dehydrogenase and antibodies for soluble interleukin-2 receptor) and urine were within the normal ranges, except for slight elevation of the C-reactive protein level (0.6 mg/dL). An HIV antibody test was negative.

**Figure 1:**
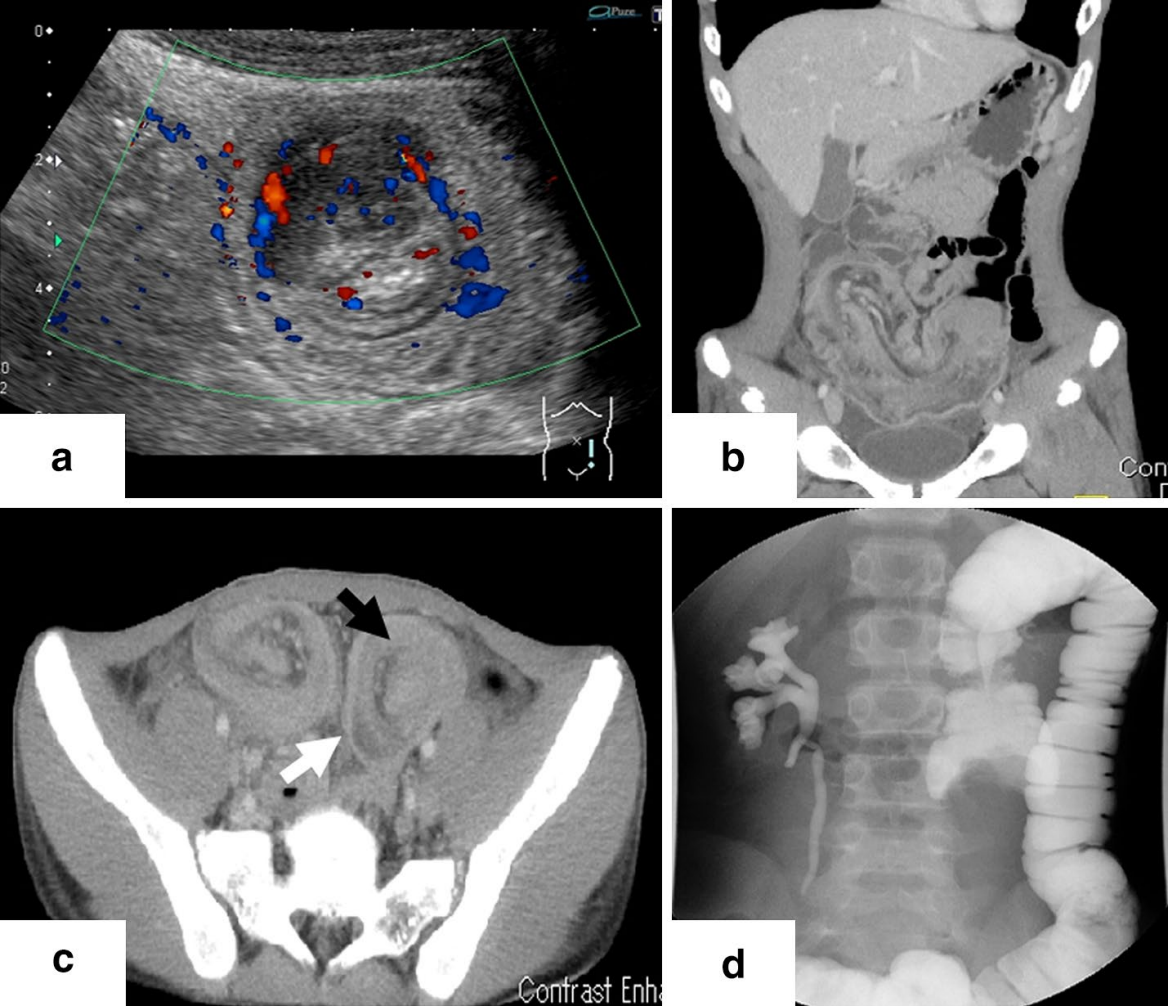
Preoperative images. **a** Ultrasonography showed the “target sign” in the lower abdomen. **b** Enhanced CT revealed ileocolic intussusception and stretched mesenteric vessels. **c** The outer wall of the area of intussusception consisted of an edematous ileum (inner layer; *black arrow*) and dilated colon (outer layer; *white arrow*). **d** An enema examination showed “crab’s claw sign” in the transverse colon.

**Figure 2:**
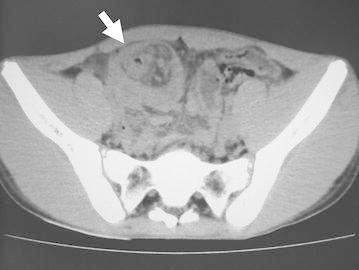
Abdominal CT at previous clinic. This CT was performed 5 weeks before he admitted to our hospital. Retrospectively, intussusception could be pointed out (*arrow*).

An ultrasonography examination of the abdomen showed a target sign at the lower abdomen, indicating the existence of intussusception (Figure 1). Enhanced computed tomography (CT) also revealed ileocolic intussusception with a suspicious looking mass (Figure 1). In addition, an examination of abdominal CT performed at the previous clinic 5 weeks earlier made us suspicious of signs of ileocolic intussusception (Figure 2). Therefore, the initial appearance of the intussusception coincided with the beginning of his symptoms 5 weeks previously. We concluded that his symptoms were due to chronic intussusception, with an organic lead point. An attempt at hydrostatic reduction with gastrografin^®^ (Bayer Yakuhin, Osaka, Japan) was not successful (Figure 1); therefore, an emergent operation was conducted.

**Figure 3:**
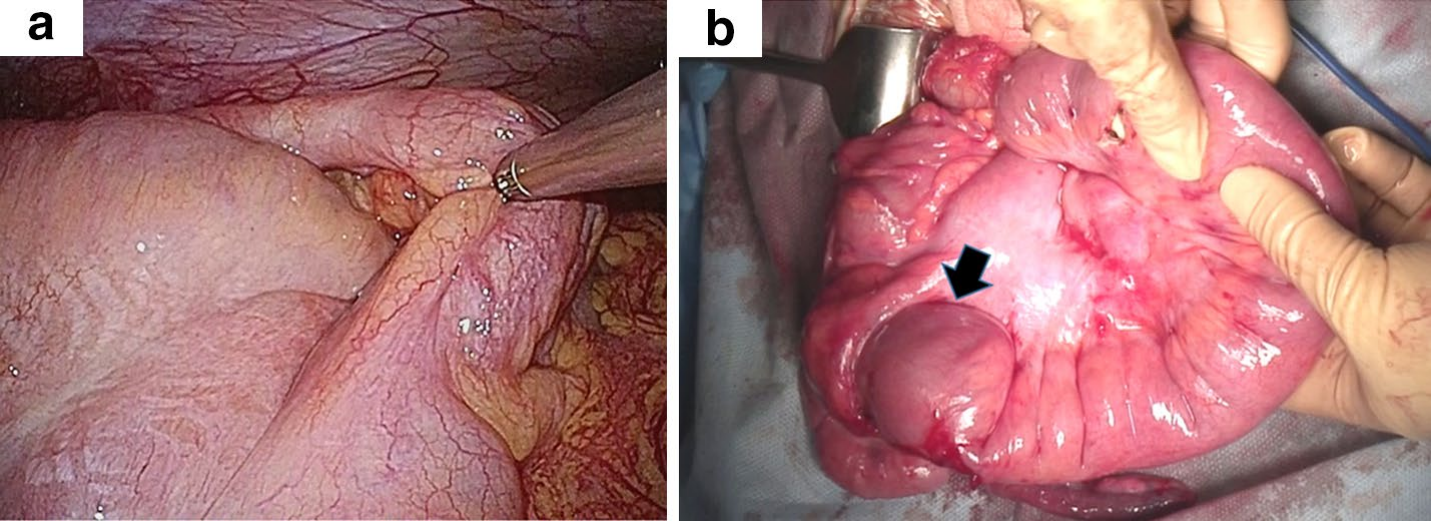
Intraoperative findings. **a** Ileocolic intussusception was confirmed. Laparoscopic reduction failed. **b** The mesentery was markedly thickened, and the ileum was edematous. Although the tumor (*arrow*) in the terminal ileum was partially reduced, manual reduction was unsuccessful. Ileocecal resection was performed.

Initially, we performed probe laparoscopy, which revealed ileocolic intussusception, serous ascites and a markedly edematous ileum and mesentery (Figure 3). We then tried laparoscopic reduction of the intussusception, which resulted in failure, and therefore converted to open surgery. However, the intussusception could not be reduced, even with the Hutchinson maneuver. The mesentery was remarkably edematous, thickened and hemorrhagic. Finally, the mesentery was dissected along with intestine, and ileocecal resection with functional end-to-end anastomosis was performed. The patient’s postoperative course was uneventful, and a cytological examination of the drained ascites fluid was negative for malignancy.

**Figure 4:**
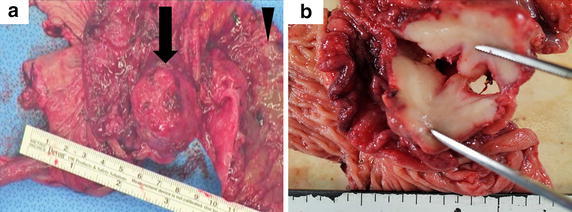
Resected specimen. **a** The tumor (*black arrow*) was located on the antimesenteric side 2 cm oral to the ileocecal valve. The mucosa of the ileum was markedly edematous and erosive (*arrowhead*). **b** The tumor was hard and contained a white section. The shape of the tumor was distorted, especially in the ileal serosa.

**Figure 5:**
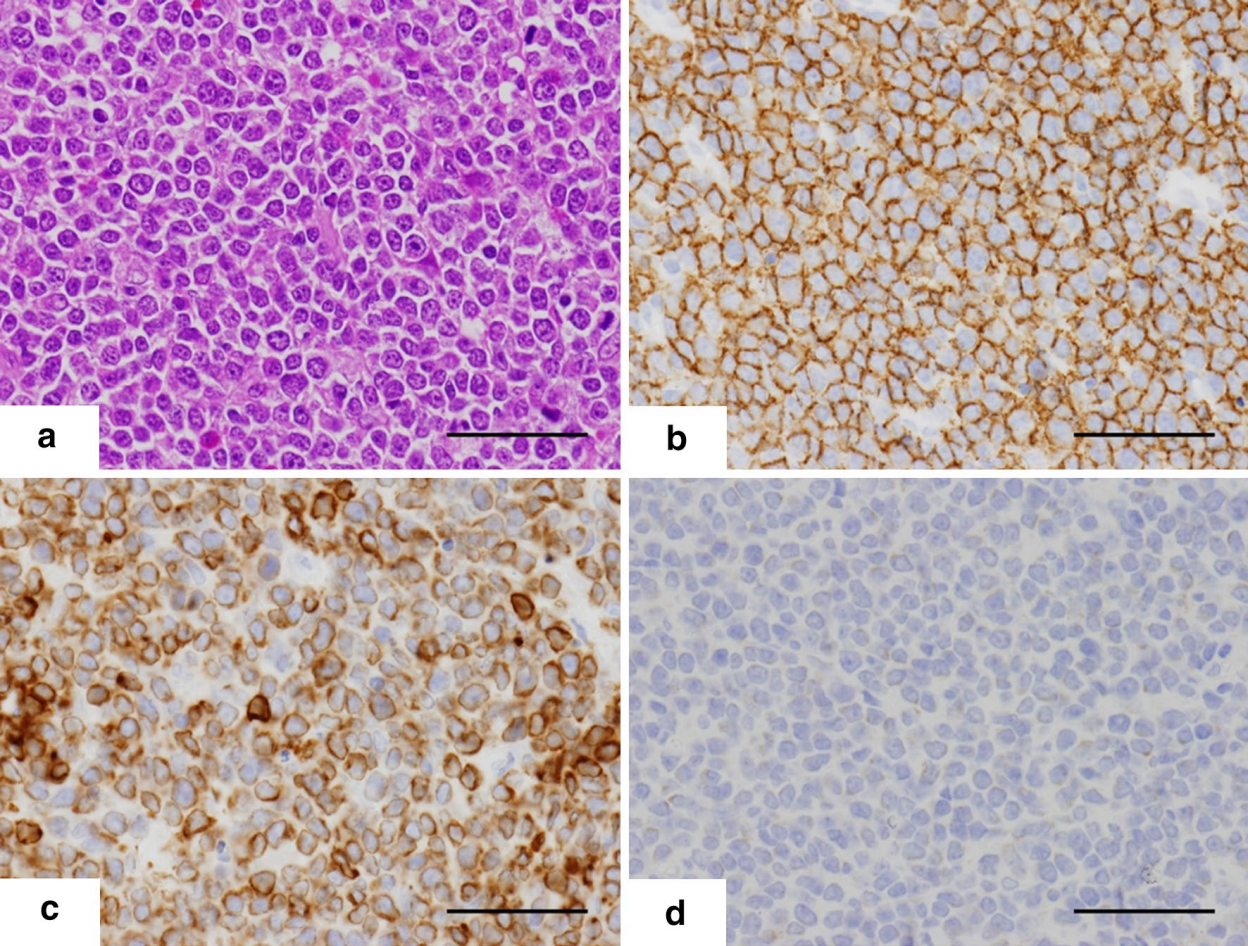
Representative histological images of the tumor cells in the resected specimen. **a** Hematoxylin–eosin (HE) stain. **b**–**d** Immunohistochemistry. **b** CD20. **c** BCL2. **d** TdT. Positive cells stained brown on immunohistochemistry. Original magnification ×400. *Bar* 50 μm.

In the resected specimen, a 3 cm submucosal firm mass was found 2 cm oral from the ileocecal valve (Figure 4). The mucosa of the terminal ileum was edematous and partially erosive. Histologically, the ileocecal mass was composed of the transmural diffuse proliferation of medium- to large-sized lymphoid cells (Figure 5a). Immunohistochemistry revealed that the tumor cells were positive for CD20 (Figure 5b), CD79a (data not shown) and Bcl-2 (Figure 5c). The tumor cells also showed positive immunostaining for CD10 and Bcl-6 in the majority and c-myc in part. In contrast, immunostaining for CD3 (data not shown), TdT (Figure 4d), CD34, CD23 and cyclin D1 was negative (data not shown). Around 70% of the tumor cells were positive for Ki-67. These findings led to a diagnosis of diffuse large B-cell lymphoma (DLBCL) of the terminal ileum. There were no obvious metastatic lesions on the postoperative FDG PET/CT scans. According to the Murphy staging system, we classified the patient as having stage II disease.

The postoperative course was uneventful, and the patient was transferred to the hematology unit of another hospital 2 weeks after the operation, at which time adjuvant chemotherapy was administered.

## Discussion

Intussusception is usually considered to be a readily diagnosable disease in terms of the susceptible age (under 2 years old) and characteristic symptoms, including intermittent abdominal pain, vomiting, bloody stools and palpable masses. The development of symptoms depends on the degree of strangulation and obstruction of the bowel.

Chronic intussusception, lasting over 14 days, is a rare condition (Rees and Lari 1976). The incidence of chronic intussusception has been reported to be 5.2% in all cases of intussusception and is higher in patients age above 1 year of age (10.3%) than in those with an age below 1 year (3.1%) (Macaulay and Moore 1955). Chronic intussusception is non-strangulated and incompletely obstructing. Non-strangulating intussusception may reduce spontaneously, progress to strangulation or remain stable (Schulman et al. 1998). In cases of ‘chronic’ intussusception, nonspecific symptoms, including diarrhea, anorexia and weight loss, may be present, in addition to typical symptoms associated with ‘acute’ intussusception (Macaulay and Moore 1955; Reijnen et al. 1989). These factors associated with chronic intussusception may make confirming the definitive diagnosis of ‘chronic’ intussusception challenging, particularly when the initial treatment is aimed to treat gastroenteritis, as described in our case (Shekhawat et al. 1992).

Two mechanisms of chronic intussusception can be assumed, that is: (1) reduction and invagination may repeat spontaneously or (2) the intussusception may remain unchanged. In the current case, the latter is an acceptable explanation because (1) we detected findings compatible with intussusception on the CT scan obtained at the former clinic, (2) the mucosa of the terminal ileum was severely erosive and edematous and (3) manual reduction was impossible even after the excision. Although achieving hydrostatic reduction of chronic intussusception is difficult (Rees and Lari 1976; Macaulay and Moore 1955), contrast enemas with or without low hydrostatic pressure may be useful for obtaining the diagnosis. In this case, we performed contrast enema with low hydrostatic pressure to obtain any information of leading point, which resulted in failure. Contrast enema was not necessarily required in previously diagnosed chronic intussusception same as our case. Because an organic lead point may frequently be present in patients with chronic intussusception, early surgical intervention should be applied (Schulman et al. 1998; Reijnen et al. 1989).

Although cases of intussusception in children are usually ‘idiopathic’, approximately 5% of patients have a pathological lead point, including Meckel’s diverticulum, duplication cysts, polyps or lymphoma (Applegate 2009). Intussusception occurring in older children and adults is accompanied by a significantly higher incidence of coexisting neoplasms (Hsiao et al. 2013). Although pathological lead points in the small intestine are usually benign, around 30% of cases are secondary to malignant lesions, including malignant lymphomas (Akbulut 2012).

The gastrointestinal tract is one of the most common extranodal sites for non-Hodgkin lymphoma. Approximately 80–90% of primary gastrointestinal tract lymphomas are of B-cell origin (Li et al. 2008). Lymphomas arising in the gastrointestinal tract present with various symptoms, including abdominal pain, anorexia, weight loss, diarrhea and ileus (Koch et al. 2001) and can be a rare pathological lead point of intussusception, as in our case (Shakya et al. 2009). Sometimes pediatric lymphomas may be related to underlying abnormalities of immunity associated with either congenital causes, infections, such as with the Epstein Barr virus, autoimmunity or inflammatory bowel diseases. In the present case, there were no clinical findings indicating innate or acquired immunodeficiency. Furthermore, using in situ hybridization, the tumor cells were found to be negative for Epstein Barr virus-encoded small RNA (data not shown).

Although there are some reports of the non-operative management of lymphoma presenting with intussusception (Lerner et al. 2011; Kang et al. 2014), the administration of chemotherapy under conditions of intussusception due to lymphoma may have the potential risk of causing tumor lysis syndrome and perforation of the intestine. If the patient may tolerate surgical intervention, resection of the affected intestine is a reasonable strategy for reducing the tumor volume and making the correct histopathological diagnosis. Gupta et al. reported that pediatric patients with Burkitt’s lymphoma presenting with intussusception often have completely resectable disease and assumed that the detection of intussusception may lead to an early diagnosis (Gupta et al. 2007). We assume that when the size of enteric lymphoma is small, the tumor tends to cause intussusception rather than obstruction and that when the tumor grows too to be large to cause invagination, the lesion may cause intestinal obstruction.

## Conclusion

The present case illustrates the diagnostic challenges of confirming ‘chronic’ intussusception in older children who present with continuous abdominal symptoms, such as diarrhea, anorexia and abdominal pain. It is important to take account of chronic intussusception regardless of age if the abdominal symptoms lasts despite the initial treatment aimed to treat gastroenteritis.

## Consent

Written informed consent was obtained from the patient’s parent for the publication of this report and any accompanying images.
